# Genetic profiling of young and aged endothelial progenitor cells in hypoxia

**DOI:** 10.1371/journal.pone.0196572

**Published:** 2018-04-30

**Authors:** Tzu-Wei Wu, Chun-Chieh Liu, Chung-Lieh Hung, Chih-Hsien Yen, Yih-Jer Wu, Li-Yu Wang, Hung-I Yeh

**Affiliations:** 1 Department of Medicine, Mackay Medical College, New Taipei City, Taiwan; 2 Section of Cardiology, Department of Internal Medicine, Mackay Memorial Hospital, Taipei City, Taiwan; Centro Cardiologico Monzino, ITALY

## Abstract

Age is a major risk factor for diseases caused by ischemic hypoxia, such as stroke and coronary artery disease. Endothelial progenitor cells (EPCs) are the major cells respond to ischemic hypoxia through angiogenesis and vascular remodeling. However, the effect of aging on EPCs and their responses to hypoxia are not well understood. CD34^+^ EPCs were isolated from healthy volunteers and aged by replicative senescence, which was to passage cells until their doubling time was twice as long as the original cells. Young and aged CD34^+^ EPCs were exposed to a hypoxic environment (1% oxygen for 48hrs) and their gene expression profiles were evaluated using gene expression array. Gene array results were confirmed using quantitative polymerase chain reaction, Western blotting, and BALB/c female athymic nude mice hindlimb ischemia model. We identified 115 differentially expressed genes in young CD34^+^ EPCs, 54 differentially expressed genes in aged CD34^+^ EPCs, and 25 common genes between normoxia and hypoxia groups. Among them, the expression of solute carrier family 2 (facilitated glucose transporter), member 1 (SLC2A1) increased the most by hypoxia in young cells. Gene set enrichment analysis indicated the pathways affected by aging and hypoxia most, including genes “response to oxygen levels” in young EPCs and genes involved “chondroitin sulfate metabolic process” in aged cells. Our study results indicate the key factors that contribute to the effects of aging on response to hypoxia in CD34^+^ EPCs. With the potential applications of EPCs in cardiovascular and other diseases, our study also provides insight on the impact of ex vivo expansion might have on EPCs.

## Introduction

Within the past millennium, the human lifespan has extended substantially because of progress in medicine. However, one of the major challenges in modern medicine is maintaining the quality of life of people undergoing the physical, psychological, and social changes associated with aging [1]. Preventing, detecting, and curing age-related diseases is essential to limit the physical aspects of aging. Age is a major risk factor for neurodegenerative diseases, such as Alzheimer’s disease, and cardiovascular diseases, such as stroke and coronary artery disease [2–5]. In Taiwan, coronary artery disease and stroke account for > 18% of mortality and are 2 of the 3 leading causes. Cardiovascular events are closely associated with metabolic syndrome [6–10], which is a group of conditions including hypertension, high blood glucose, and hyperlipidemia. As the metabolic syndrome progresses, blood vessel thickness, measured in common carotid intima-media thickness, increases [11–14], eventually resulting in the blockage of blood vessels and local ischemic hypoxia, causing life-threatening cardiovascular events [15–18].

Hypoxia is a pathological condition in which the body as a whole (generalized hypoxia) or a region of the body (tissue hypoxia) is deprived of adequate oxygen supply. Hypoxia usually indicated an oxygen concentration under 1% in our tissue while the atmospheric oxygen concentration is about 20%, and the physiological oxygen concentration is around 5% [19,20]. Among the various types of hypoxia, ischemic hypoxia is caused by a local restriction in the flow of well-oxygenated blood. Ischemic hypoxic tissue typically recovers through angiogenesis and vascular remodeling; however, this recovery ability decreases with age [21].

Endothelial progenitor cells (EPCs) are a population of circulatory cells that conduct angiogenesis and vascular remodeling through their ability to differentiate into endothelial cells and form blood vessels [22]. EPCs were first characterized as a purified population of CD34-expressing cells isolated from the blood of adult mice, which demonstrated the ability to differentiate into endothelial cells in vitro [23]. Various cytokines, growth factors, hormones, and ischemic conditions can cause EPCs to be mobilized primarily from bone marrow into the peripheral circulation, ultimately homing to regions of angiogenesis [24,25]. Circulating EPCs repaired damaged blood vessels after a myocardial infarction, and high levels of circulating EPCs were predictive of favorable outcome, with patients experiencing few repeat heart attacks [26].

In ex vivo culture, EPCs undergo replicative senescence [27]. It is possible that aged patients suffer from ischemic disorders to a greater extent than young patients do because of reduced ability for angiogenesis and vascular remodeling, resulting from changes in EPCs capabilities to respond to hypoxia [28]. In this study, to elucidate the possible mechanisms underlying aging-related diseases, we investigated the gene profiles of young and aged CD34^+^ EPCs in hypoxia and validated our results in cell and animal models.

## Materials and methods

### Isolation and culture of endothelial progenitor cells

This study was a part of the MAGNET (Mitochondria-AGing in NorthErn Taiwan) study [29]. The study protocol was reviewed and approved by the Institution Review Board of Mackay Medicine College (No. P990001). An invitation letter describing the study objectives and methods was distributed to healthy volunteers aged 20–40 years. Written consent was obtained from all volunteers. A basic physical examination was performed on each volunteer, including a blood pressure check, before 80 mL of peripheral blood were collected. Individuals with high (SBP≧140 or DPB≧90) or low (SBP≦90 or DPB≦60) blood pressure, or any self-reported health conditions were excluded. EPCs were isolated as described in our previous publication [30]. In short, peripheral blood mononuclear cells were fractionated by centrifugation on a Ficoll-Paque Plus (17-1440-02, GE Healthcare, Amersham, USA). CD34^+^ cells were selected by passing through a column containing CD34 antibody-coated MACS MicroBeads (130-046-702, Miltenyi Biotec, Bergisch Gladbach, Germany). Isolated cells were seeded on fibronectin-coated dishes. Adherent cells were extensively washed on day 4 to remove unattached cells, and cultured in fresh MV2 medium (C22022, PromoCell, Heidelberg, Germany) containing 20% fetal bovine serum. As described in the supplementary information in our previous publication, CD34^+^ cells isolated with this protocol demonstrated several endothelial progenitor cell properties including the ability to take up Dil-Ac-LDL, positive expression of UEA-1, and the ability to form tube-like structures in vitro [30].

### Hypoxic conditions

EPCs were cultured in a CO_2_ Cell Culture Incubator (SANYO MCO-5AC, San Diego, USA) under atmospheric oxygen (20% O_2_) and hypoxic conditions (1% O_2_) for designated durations.

### Determination of cell doubling time

Cells were cultured in 24-well plates and 3-(4,5-dimethylthiazol-2-yl)-2,5-diphenyltetrazolium bromide (MTT) solution (SI-M5655, Sigma-Aldrich, St. Louis, MO) was added to the medium to a final concentration of 0.5 mg/mL. Cells were incubated with MTT for 2 hours at 37°C. The MTT crystals were dissolved in dimethyl sulfoxide, and the optical density at a wavelength of 570 nm was measured by using a microplate reader FlexStation 3 (Molecular Devices, CA).

### Evaluation of senescence by using β-galactosidase staining

The number of senescent cells was determined using a β-galactosidase (β-gal) staining kit (9860, Cell Signaling Technology Inc, Danvers, MA) according to the manufacturer’s protocol. The culture medium was removed from EPCs, which were then washed once with phosphate buffered saline (PBS). The washed EPCs were incubated in a 1× fixation solution for 15 minutes at room temperature and then washed twice with PBS before incubating with a staining solution at 37°C (without carbon dioxide (CO_2_) control) overnight. Images of cells were captured under a light microscope and the number of positive cells was counted in a blinded manner.

### Western blotting

Total proteins or fractionated proteins were separated by sodium dodecyl sulfate polyacrylamide gel electrophoresis and transferred to polyvinylidene difluoride membranes (Millipore Corp., Bedford, MA, USA). The membranes were blocked with 3% milk in wash solution (0.5% Tween 20 in PBS) and probed with primary antibodies (anti-VEGF, 1:1000, Santa Cruz Biotechnology Inc, Delaware Ave, Santa Cruz; Anti- SLC2A1, 1:5000, ab40084, Abcam, Cambridge, USA) at 4°C overnight. The blots were normalized by reprobing with β-actin (1:3000, Cell Signaling, Beverly, MA, USA). The membranes were incubated with horseradish peroxidase-conjugated secondary antibodies (1:5000, Vector Laboratories, Burlingame, CA) for 1 hour at room temperature, and results were visualized using Immobilon Western Chemiluminescent HRP Substrate (Millipore Corporation, Billerica, MA, USA). Results were captured by the UVP BioSpectrum 500 Imaging System. Relative amounts of protein were quantified by optical density analysis using VisionWorksLs Image Acquisition and Analysis software (Upland, CA, USA).

### Gene expression microarray and gene set enrichment analysis

Gene expression patterns in CD34^+^ EPC in 4 conditions (young and aged cells; in normoxia and hypoxia) were measured using gene expression microarray (HumanHT-12 v4 Expression BeadChip, Illumina, Inc, San Diego, CA). Three CD34^+^ EPCs clones isolated from different donors were used. Young and aged CD34^+^ EPCs were cultured in normoxia or hypoxia (1% O_2_) for 48 hours. Total RNA samples were isolated from CD34^+^ EPCs by using Trizol. The result was analyzed based on the method by Li et al. [31]. In short, quantile normalization was performed on raw intensity readings from all samples. Followed by ANOVA analysis to each gene (one-way ANOVA for genes with one probe on the array; two-way ANOVA for genes with two probes on the array). Genes with ≥ 1.5- or ≤ 0.67-fold changes, and *P* < .05, were selected. Microarray chip processing services were provided by Genetech Biotech Co., Ltd, Taiwan. Genes with significant changes in each gene set were functionally analyzed using hypergeometric testing and grouped into functional gene ontology (GO) groups.

### Real-time polymerase chain reaction

Total RNAs were isolated from CD34^+^ EPCs by using TRIzol (Cat. No. 15596–026, Invitrogen, Grand Island, NY) according to the manufacturer’s instructions. RNAs were reverse-transcripted to cDNAs by using a poly-T primer and subjected to polymerase chain reaction (PCR) analysis. For quantitative PCR (Q-PCR) analysis, cDNA was amplified using gene specific probes and Taqman Universal Master Mix (Invitrogen, Grand Island, NY) according to the manufacturer’s protocol. The Q-PCR analysis was performed in triplicate by using a real-time PCR machine (ABI 7900, Applied Biosystems, Grand Island, NY). The gene expression of each sample was normalized to the expression of 18S.

### Hindlimb ischemic animal model and injection of EPCs

The work was conducted in accordance with the Republic of China Animal Protection Law (Scientific Application of Animals) of 1998. The protocol was approved by Institutional Animal Care and Use Committee, Mackay Medical College (Protocol number A10200212). We attest that all efforts were made to minimize the number of animals used and their suffering. Totally three BALB/c female athymic nude mice, aged 8 weeks and weighing 18 to 22 g, were used. Mice were housed in 315×230×160 mm cages with bedding under controlled temperature (22°C), humidity, and light (14 hour light: 10 hour dark) conditions. Standard laboratory mouse diet and water were available *ad libitum*. To create the hindlimb ischemia model, the right femoral artery and vein were ligated and cut from just above the deep femoral arteries to the popliteal artery and vein. During recovery from the anesthesia, body temperature was maintained at 37°C with a heating lamp. Twenty-four hours after surgery, animals were injected intramuscularly with 2×10^5^ CD34^+^ EPCs in 50 μL of saline into both thighs and calfs. Forty-eight hours later, animals were euthanized by intraperitoneal injection of an overdose of pentobarbital. Calf muscles were dissected and prepared for immunohistochemstry examination.

### Immunoflurescence staining

CD34^+^ EPCs grown on coverslips were fixed with 4% paraformaldehyde for 10 minutes, permeabilized with 0.2% Triton X-100 for 30 minutes and blocked with 2% bovine serum albumin for 1 hour at 4°C before primary antibody incubation. The slides were incubated with the anti-SLC2A1 primary antibody (1:200, ab128033, Abcam, UK) at RT for 2 hour. After washing three times with PBS the slides were incubated for 1 hour with DyLight 488-Conjugated secondary antibody (1:200, Jackson Laboratory, USA) before counterstained with DAPI (Invitrogen).

Calf muscles were placed in 30% sucrose-PBS for 24 hours, bisected at the middle level, mounted in Tissue-Tek O.C.T compound (Cat. No. 4583, Sakura Finetechnical, Tokyo, Japan) with liquid nitrogen-cooled-2-methylbutane. A series of adjacent 5-mm-thick frozen sections were cut from each muscle in the slide. The sections were fixed in 4% paraformaldehyde for 10 min, washed briefly with PBS, and incubated with MOM kit (Vector, Burlingame, CA, USA) to blocked tissue-nonspecific antigen. The samples were incubated with the anti-SLC2A1 antibody (1:200, ab128033, Abcam, UK) and anti-human-specific nuclear antigen antibody (1:50; Chemicon) at RT for 2 hour. After washing three times with PBS the samples were incubated for 1 hour with DyLight 488 or DyLight 594 secondary antibodies (1:200, Jackson Laboratory, USA) and counterstained with DAPI (Invitrogen). Finally, the slides were with ProLong Diamond Antifade mountant (Cat No. P36961, Invitrogen, USA). The images were taken with Zeiss Axio Z1 fluorescence microscope with Axiovision software (Carl Zeiss AG, Germany) and analyzed with Metamorph software (Molecular Device, USA).

### Statistical analysis

All statistical analyses were performed using SAS 9.1 (SAS Institute Inc, Cary, NC, USA). ANOVA plus meta-analysis was used to determine the significance of differences in the mean levels of gene expression between groups. *P* < .05 was considered significant.

## Results

### Replicative senescence of CD34^+^ EPCs induced by in vitro passage

**Fig 1:**
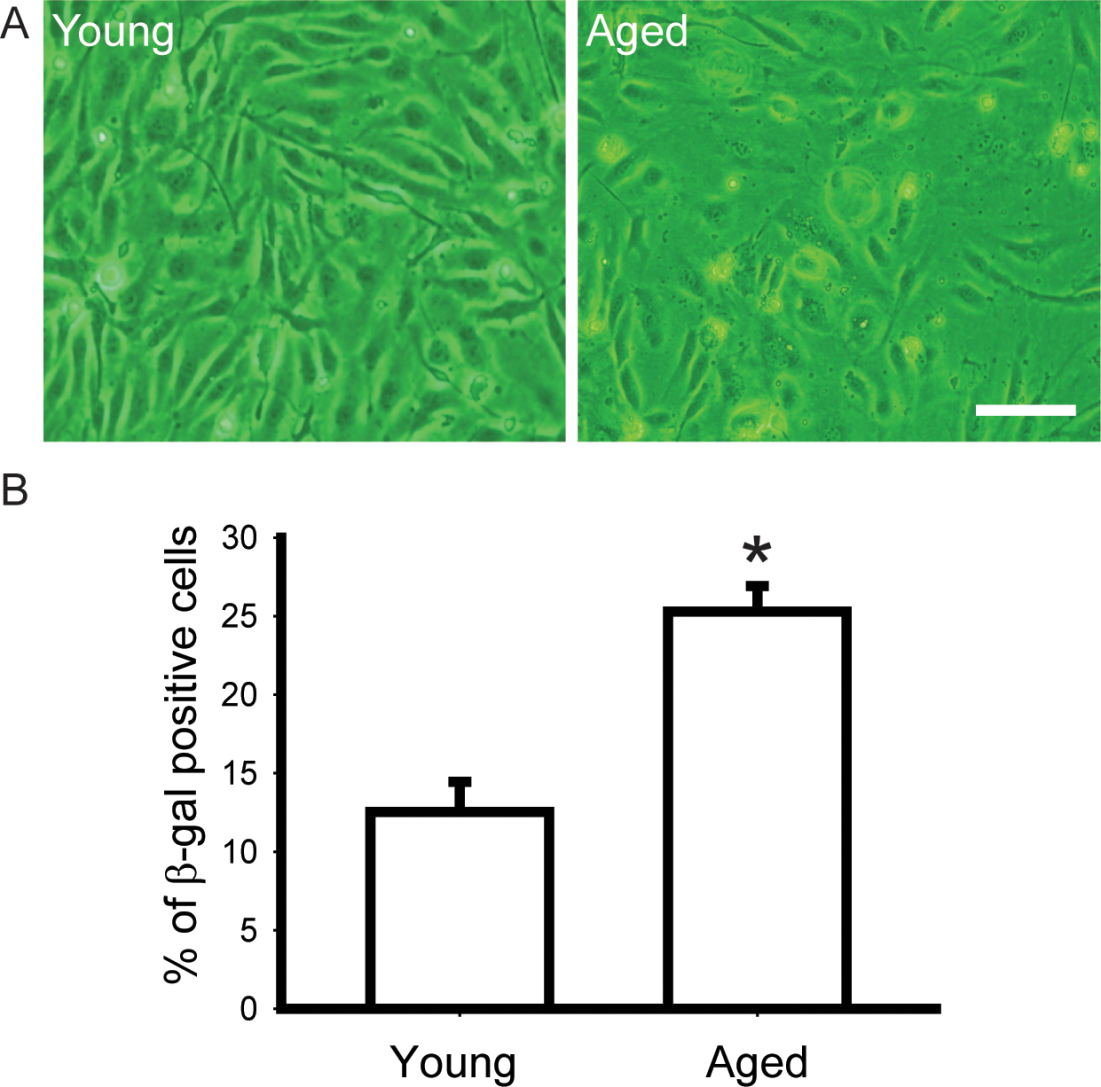
Isolation and aging of CD34^+^ EPC. Isolated CD34^+^ EPC were subcultured in vitro and signs of senescence showed after around 10 passages. Replicative senescence resulted in (A) morphological changes (Bar = 10μm), (B) up-regulation of senescence marker (P < .01).

We isolated and cultured CD34^+^ EPCs from different healthy donors; cells isolated from one blood sample of one donor were cultured as one EPCs clone. We passaged the cells when confluent until their doubling time doubled (aged EPCs) compared with the original cells (young EPCs), as indicated by MTT assay (Fig 1C). The passage numbers of young and aged CD34^+^ EPCs used in this analysis were 7 and 19; 6 and 19; 7 and 12; respectively. We observed morphological differences between the young and aged CD34^+^ EPCs. Young CD34^+^ EPCs were cobblestone appearance with high cell-cell contact in culture (Fig 1A, left), whereas aged CD34^+^ EPCs were slender and flat, with irregular edges and low cell-cell contact (Fig 1A, right). The two-fold increase of doubling time required 10.3±2.2 passages. We generated young and aged CD34^+^ EPCs from the same CD34^+^ EPCs clone for subsequent experiments, and detected the proportion of senescent cells in a population by using β-gal staining. We observed that the aged CD34^+^ EPCs population contained more than twice the percentage of senescent cells compared with the young CD34^+^ EPCs population (Fig 1B).

### Determination of hypoxic conditions

**Fig 2:**
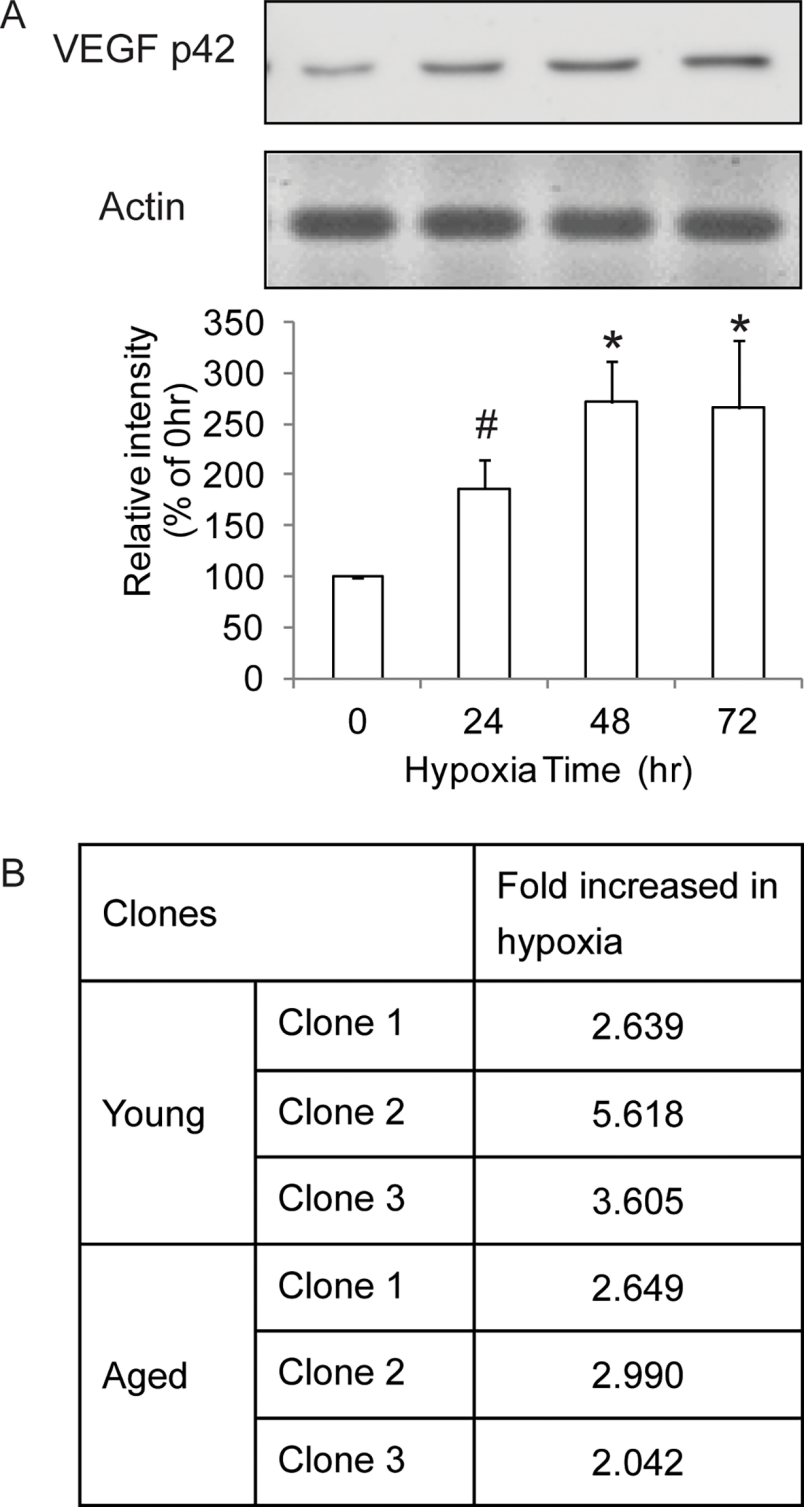
Determination of hypoxia time. (A) VEGF Western blot time course showed that VEGF was upregulated within 24 hours and reached a plateau in 48 hours. N = 3; * P< .01; # P< .05 (B) VEGF upregulation was confirmed by expression microarray in 3 EPC clones.

To determine the duration of culture in 1% oxygen that maximally provoked CD34^+^ EPCs against hypoxia, as judged by the expression of VEGF, a marker of hypoxia, cells were incubated for different periods of time in 1% oxygen followed by Western blot analysis. The results showed that, in young CD34^+^ EPCs, the expression level of VEGF protein increased by approximately 80% after 24 hours of hypoxia, continued to increase by 48 hours (> 2.5-fold), and remained at the same level after 72 hours of hypoxia (Fig 2A). Based on the expression level of VEGF protein, we applied 1% oxygen treatment for 48 hours in gene profiling analyses. Our microarray results confirmed the upregulation (≥ 2-fold increases) of VEGF in 3 clones of EPCs subject to hypoxia, regardless of young or old cells (Fig 2B).

### Expression array of young and aged CD34^+^ EPC in response to hypoxia

In the same 3 clones of CD34^+^ EPCs subject to hypoxia, we identified 115 hypoxia-induced differentially expressed genes in young CD34^+^ EPCs (S1 and S3 Tables), 54 hypoxia-induced differentially expressed genes in aged CD34^+^ EPCs (S2 and S3 Tables), and 25 hypoxia-induced differentially expressed genes in both young and aged CD34^+^ EPCs (S3 Table). Table 1 lists the hypoxia-induced differentially expressed genes and their fold changes in young and aged CD34^+^ EPCs, as well as the ratio of changes between young and aged CD34^+^ EPCs. The gene associated with the greatest increase in expression in young CD34^+^ EPCs in response to hypoxia was solute carrier family 2 (facilitated glucose transporter), member 1 (SLC2A1; 4.477-fold increase). SLC2A1 was also the gene associated with the greatest upregulation of expression in aged CD34^+^ EPCs exposed to hypoxia (4.497-fold increase), indicating its role in EPCs response to hypoxia. When we compared the differences in fold changes in gene expression between young and aged CD34^+^ EPCs, we observed that solute carrier organic anion transporter family, member 2A1 (SLCO2A1) was associated with the greatest differences in gene expression. In hypoxic conditions, SLCO2A1 expression was reduced to 20% in young EPCs and 80% in aged CD34^+^ EPCs.

**Table 1:** All differentially expressed genes.

Gene Symbol	Gene Description	fold change (p-value), Young (hy/normal)	fold change (p-value), Old (hy/normal)	fold change (Old/Young)
SLCO2A1	solute carrier organic anion transporter family, member 2A1 (SLCO2A1)	0.276(0.0284)	0.831(0.1742)	3.002
ID2	inhibitor of DNA binding 2, dominant negative helix-loop-helix protein (ID2)	0.645(0.0145)	1.358(0.2341)	2.105
FAM107A	family with sequence similarity 107, member A (FAM107A), transcript variant 2	1.043(0.9038)	2.178(0.0160)	2.088
DNASE1L3	deoxyribonuclease I-like 3 (DNASE1L3)	0.870(0.4691)	1.651(0.0115)	1.896
LHFPL2	lipoma HMGIC fusion partner-like 2 (LHFPL2)	0.615(0.0267)	1.104(0.4028)	1.795
ABCA1	ATP-binding cassette, sub-family A (ABC1), member 1 (ABCA1)	0.876(0.1384)	1.543(0.0110)	1.761
NQO1	NAD(P)H dehydrogenase, quinone 1 (NQO1), transcript variant 1	0.242(0.0011)	0.409(0.1437)	1.691
DDIT4	DNA-damage-inducible transcript 4 (DDIT4)	1.346(0.0430)	2.237(0.0228)	1.661
LGALS9	lectin, galactoside-binding, soluble, 9 (LGALS9), transcript variant 1	0.614(0.0352)	1.017(0.7327)	1.654
ANKRD37	ankyrin repeat domain 37 (ANKRD37)	2.954(0.0206)	4.870(0.0162)	1.648
SNORD13	small nucleolar RNA, C/D box 13 (SNORD13), small nucleolar RNA.	1.147(0.6815)	1.771(0.0392)	1.544
ITM2B	integral membrane protein 2B (ITM2B)	1.031(0.7240)	1.581(0.0040)	1.533
HSPA1B	heat shock 70kDa protein 1B (HSPA1B)	0.609(0.0140)	0.929(0.7170)	1.523
MALL	mal, T-cell differentiation protein-like (MALL)	0.611(0.0476)	0.919(0.7652)	1.504
LOC100129759	similar to PNAS-117 (LOC100129759)	0.657(0.0136)	0.988(0.9303)	1.502
FLRT2	fibronectin leucine rich transmembrane protein 2 (FLRT2)	0.418(0.0151)	0.628(0.1131)	1.500
SESN1	sestrin 1 (SESN1)	0.644(0.0089)	0.964(0.3835)	1.497
VEGFC	vascular endothelial growth factor C (VEGFC)	3.778(0.0063)	2.515(0.0017)	0.665
COL4A2	collagen, type IV, alpha 2 (COL4A2)	2.105(0.0036)	1.400(0.0208)	0.665
HS.10862	cDNA: FLJ23313 fis, clone HEP11919	3.696(0.0073)	2.449(0.0009)	0.662
SLC16A3	solute carrier family 16, member 3 (monocarboxylic acid transporter 4) (SLC16A3), transcript variant 2	1.798(0.0222)	1.171(0.6710)	0.651
PGF	placental growth factor (PGF)	1.767(0.0401)	1.146(0.6088)	0.648
EFEMP2	EGF-containing fibulin-like extracellular matrix protein 2 (EFEMP2)	2.009(0.0036)	1.293(0.1873)	0.643
GGT3	misc_RNA (GGT3)	2.057(0.0014)	1.320(0.0960)	0.641
NOX4	NADPH oxidase 4 (NOX4), transcript variant 1	1.960(0.0484)	1.253(0.2460)	0.639
BNIP3	BCL2/adenovirus E1B 19kDa interacting protein 3 (BNIP3), nuclear gene encoding mitochondrial protein	1.843(0.0201)	1.164(0.3878)	0.631
ECGF1	endothelial cell growth factor 1 (platelet-derived) (ECGF1)	2.188(0.0204)	1.356(0.0338)	0.620
LPXN	leupaxin (LPXN)	0.809(0.0559)	0.498(0.0241)	0.615
AK1	adenylate kinase 1 (AK1)	1.860(0.0224)	1.140(0.5647)	0.613
ALDOC	aldolase C, fructose-bisphosphate (ALDOC)	2.386(0.0004)	1.464(0.0244)	0.613
HYI	hydroxypyruvate isomerase homolog (E. coli) (HYI)	1.861(0.0243)	1.135(0.1143)	0.610
SELE	selectin E (endothelial adhesion molecule 1) (SELE)	0.979(0.7610)	0.590(0.0314)	0.603
AXUD1	AXIN1 up-regulated 1 (AXUD1)	1.913(0.0257)	1.144(0.5125)	0.598
SPOCK1	sparc/osteonectin, cwcv and kazal-like domains proteoglycan (testican) 1 (SPOCK1)	2.917(0.0403)	1.704(0.0226)	0.584
SERPINE1	serpin peptidase inhibitor, clade E (nexin, plasminogen activator inhibitor type 1), member 1 (SERPINE1)	2.290(0.0327)	1.338(0.2615)	0.584
TGFBI	transforming growth factor, beta-induced, 68kDa (TGFBI)	3.258(0.0002)	1.897(0.2238)	0.582
SRPX2	sushi-repeat-containing protein, X-linked 2 (SRPX2)	1.767(0.0120)	1.028(0.9488)	0.581
DUSP1	dual specificity phosphatase 1 (DUSP1)	2.387(0.0062)	1.387(0.0918)	0.580
TFPI2	tissue factor pathway inhibitor 2 (TFPI2)	1.097(0.6249)	0.633(0.0156)	0.577
BHLHB2	basic helix-loop-helix domain containing, class B, 2 (BHLHB2)	3.927(0.0037)	2.146(0.0007)	0.546
MSMP	microseminoprotein, prostate associated (MSMP)	1.135(0.5134)	0.604(0.0043)	0.532
TAGLN	transgelin (TAGLN), transcript variant 1	4.006(0.0024)	2.088(0.3950)	0.521
SNCAIP	synuclein, alpha interacting protein (SNCAIP)	2.080(0.0291)	1.078(0.0217)	0.518
ADSSL1	adenylosuccinate synthase like 1 (ADSSL1), transcript variant 2	1.818(7.199e-06)	0.935(0.3896)	0.514
LOC646723	similar to Keratin, type I cytoskeletal 18 (Cytokeratin-18) (CK-18) (Keratin-18) (K18) (LOC646723)	0.919(0.6277)	0.455(0.0495)	0.495
C13ORF15	chromosome 13 open reading frame 15 (C13orf15)	2.234(0.0185)	1.082(0.7791)	0.484
ERRFI1	ERBB receptor feedback inhibitor 1 (ERRFI1)	2.524(0.1770)	3.665(0.0200)	1.452
C17ORF79	chromosome 17 open reading frame 79 (C17orf79)	0.641(0.0278)	0.900(0.1720)	1.404
BTG2	BTG family, member 2 (BTG2)	0.660(0.0292)	0.921(0.6923)	1.395
LFNG	LFNG O-fucosylpeptide 3-beta-N-acetylglucosaminyltransferase (LFNG), transcript variant 1	0.604(0.0355)	0.842(0.0621)	1.393
PLIN5	perilipin 5 (PLIN5)	1.100(0.6499)	1.527(0.0385)	1.388
TGFBR3	transforming growth factor, beta receptor III (TGFBR3)	0.621(0.0331)	0.854(0.0976)	1.373
CXCL16	chemokine (C-X-C motif) ligand 16 (CXCL16)	0.580(0.0463)	0.794(0.0575)	1.368
AIF1L	allograft inflammatory factor 1-like (AIF1L), transcript variant 1	0.606(0.0046)	0.829(0.6684)	1.367
LOC375295	hypothetical gene supported by BC013438 (LOC375295)	1.474(0.1006)	1.995(0.0085)	1.353
DUXAP3	double homeobox A pseudogene 3 (DUXAP3) on chromosome 10.	1.206(0.2747)	1.633(0.0103)	1.353
MGC16121	hypothetical protein MGC16121 (MGC16121)	1.838(0.0415)	2.470(0.0164)	1.343
EPAS1	endothelial PAS domain protein 1 (EPAS1)	0.501(0.0081)	0.664(0.0049)	1.324
SYNCRIP	synaptotagmin binding, cytoplasmic RNA interacting protein (SYNCRIP)	0.652(0.0351)	0.856(0.0291)	1.313
LOC644033	similar to similar to RPL23AP7 protein (LOC644033)	0.639(0.0455)	0.838(0.2685)	1.310
ADM	adrenomedullin (ADM)	2.925(0.0201)	3.793(0.0029)	1.296
LOC441087	hypothetical gene supported by AK125735 (LOC441087)	1.188(0.1521)	1.539(0.0013)	1.294
PRRG1	proline rich Gla (G-carboxyglutamic acid) 1 (PRRG1)	0.650(0.0101)	0.839(0.3646)	1.291
GPR126	G protein-coupled receptor 126 (GPR126), transcript variant a2	1.468(0.4152)	1.879(0.0078)	1.279
SEMA4B	sema domain, immunoglobulin domain (Ig), transmembrane domain (TM) and short cytoplasmic domain, (semaphorin) 4B (SEMA4B), transcript variant 1	1.660(0.0151)	2.103(0.0374)	1.266
VCAN	versican (VCAN)	1.948(0.0784)	2.429(0.0028)	1.246
AKR1B1	aldo-keto reductase family 1, member B1 (aldose reductase) (AKR1B1)	0.631(0.0211)	0.785(0.1894)	1.243
CHD1L	chromodomain helicase DNA binding protein 1-like (CHD1L)	1.254(0.1101)	1.549(0.0440)	1.235
SDC4	syndecan 4 (SDC4)	1.315(0.2486)	1.622(0.0150)	1.233
SEC11C	SEC11 homolog C (S. cerevisiae) (SEC11C)	0.632(0.0007)	0.775(0.1333)	1.225
LOC643031	similar to NADH dehydrogenase subunit 5 (LOC643031)	1.399(0.4600)	1.687(0.0241)	1.206
NOS3	nitric oxide synthase 3 (endothelial cell) (NOS3)	0.589(0.0052)	0.706(0.2137)	1.198
SQSTM1	sequestosome 1 (SQSTM1)	0.586(0.0151)	0.666(0.0765)	1.136
CEBPD	CCAAT/enhancer binding protein (C/EBP), delta (CEBPD)	1.930(0.0123)	2.192(0.0133)	1.135
FABP4	fatty acid binding protein 4, adipocyte (FABP4)	0.494(0.0105)	0.560(0.0273)	1.134
CHST15	carbohydrate (N-acetylgalactosamine 4-sulfate 6-O) sulfotransferase 15 (CHST15)	1.343(0.0188)	1.523(0.0085)	1.133
APLN	apelin (APLN)	1.854(0.1546)	2.067(0.0458)	1.114
FAM124B	family with sequence similarity 124B (FAM124B), transcript variant 2	0.606(0.0049)	0.674(0.0432)	1.112
NDRG1	N-myc downstream regulated gene 1 (NDRG1)	1.613(0.0384)	1.748(0.0058)	1.083
TFRC	transferrin receptor (p90, CD71) (TFRC)	0.462(0.0143)	0.499(0.1856)	1.078
LYVE1	lymphatic vessel endothelial hyaluronan receptor 1 (LYVE1)	0.318(0.0434)	0.343(0.1687)	1.078
LMO2	LIM domain only 2 (rhombotin-like 1) (LMO2)	0.615(0.0102)	0.663(0.0519)	1.077
LOX	lysyl oxidase (LOX)	2.711(0.0071)	2.898(0.1194)	1.068
HMOX1	heme oxygenase (decycling) 1 (HMOX1)	0.346(0.0310)	0.369(0.0398)	1.064
ZNF323	zinc finger protein 323 (ZNF323), transcript variant 1	0.629(0.0008)	0.666(0.0622)	1.058
LDB2	LIM domain binding 2 (LDB2)	0.479(0.0168)	0.495(0.0753)	1.035
LOC647886	misc_RNA (LOC647886)	1.613(0.0217)	1.657(0.0131)	1.027
HTRA1	HtrA serine peptidase 1 (HTRA1)	1.703(0.0048)	1.749(0.0129)	1.026
SLC2A1	solute carrier family 2 (facilitated glucose transporter), member 1 (SLC2A1)	4.477(0.0022)	4.497(0.0045)	1.004
PDIA5	protein disulfide isomerase family A, member 5 (PDIA5)	1.521(0.0255)	1.505(0.0167)	0.989
ENO2	enolase 2 (gamma, neuronal) (ENO2)	1.852(0.0375)	1.830(0.0303)	0.987
ODC1	ornithine decarboxylase 1 (ODC1)	0.622(0.0475)	0.609(0.0681)	0.979
ERO1L	ERO1-like (S. cerevisiae) (ERO1L)	1.848(0.0068)	1.793(0.1016)	0.969
RASGRP3	RAS guanyl releasing protein 3 (calcium and DAG-regulated) (RASGRP3)	0.652(0.0344)	0.611(0.2326)	0.938
TOMM34	translocase of outer mitochondrial membrane 34 (TOMM34), nuclear gene encoding mitochondrial protein	0.686(0.0144)	0.636(0.0014)	0.927
EPB41L3	erythrocyte membrane protein band 4.1-like 3 (EPB41L3)	1.658(0.0406)	1.518(0.0152)	0.915
COL5A1	collagen, type V, alpha 1 (COL5A1)	1.812(0.0216)	1.650(0.1530)	0.910
NAV1	neuron navigator 1 (NAV1)	2.073(0.0009)	1.884(0.0078)	0.908
KANK1	KN motif and ankyrin repeat domains 1 (KANK1), transcript variant 1	1.687(0.0322)	1.520(0.0564)	0.900
POP1	processing of precursor 1, ribonuclease P/MRP subunit (S. cerevisiae) (POP1)	0.706(0.3091)	0.633(0.0418)	0.896
LOC441763	hypothetical LOC441763 (LOC441763)	1.619(0.0133)	1.445(0.2423)	0.892
NOL6	nucleolar protein family 6 (RNA-associated) (NOL6), transcript variant alpha	0.745(0.0145)	0.665(0.0488)	0.892
TMEM91	transmembrane protein 91 (TMEM91)	1.563(0.0125)	1.366(0.0652)	0.873
GDF15	growth differentiation factor 15 (GDF15)	0.504(0.0282)	0.439(0.0101)	0.871
FER1L4	fer-1-like 4 (C. elegans) (FER1L4) on chromosome 20.	2.108(0.0313)	1.831(0.0569)	0.868
NACC2	NACC family member 2, BEN and BTB (POZ) domain containing (NACC2)	0.623(0.0009)	0.537(0.0593)	0.862
CRELD1	cysteine-rich with EGF-like domains 1 (CRELD1), transcript variant 3	1.602(0.0194)	1.364(0.1157)	0.851
GPX1	glutathione peroxidase 1 (GPX1), transcript variant 2	0.701(0.2286)	0.595(0.0032)	0.848
MTHFD1L	methylenetetrahydrofolate dehydrogenase (NADP+ dependent) 1-like (MTHFD1L)	1.606(0.0243)	1.361(0.2833)	0.847
LOC644237	misc_RNA (LOC644237)	1.674(0.0033)	1.405(0.0056)	0.839
PTPRB	protein tyrosine phosphatase, receptor type, B (PTPRB)	1.682(0.0373)	1.402(0.0417)	0.833
SLC2A3	solute carrier family 2 (facilitated glucose transporter), member 3 (SLC2A3)	3.024(0.0145)	2.491(0.0144)	0.823
PLOD1	procollagen-lysine 1, 2-oxoglutarate 5-dioxygenase 1 (PLOD1)	1.567(0.0295)	1.290(0.5383)	0.823
JUN	jun oncogene (JUN)	1.569(0.0445)	1.289(0.1569)	0.821
DPYSL3	dihydropyrimidinase-like 3 (DPYSL3)	1.625(0.0164)	1.322(0.0703)	0.813
FBLN7	fibulin 7 (FBLN7)	1.718(0.0257)	1.388(0.1189)	0.807
GADD45B	growth arrest and DNA-damage-inducible, beta (GADD45B)	2.025(0.0209)	1.633(0.0131)	0.806
GAPDHL6	glyceraldehyde-3-phosphate dehydrogenase-like 6 (GAPDHL6)	1.707(0.0111)	1.359(0.0045)	0.796
NOTCH4	Notch homolog 4 (Drosophila) (NOTCH4)	1.517(0.0099)	1.204(0.2134)	0.793
VLDLR	very low density lipoprotein receptor (VLDLR), transcript variant 1	1.953(0.1747)	1.546(0.0278)	0.791
LOC732007	similar to Phosphoglycerate mutase 1 (Phosphoglycerate mutase isozyme B) (PGAM-B) (BPG-dependent PGAM 1) (LOC732007)	1.516(0.0379)	1.197(0.1961)	0.789
TNFAIP8L3	tumor necrosis factor, alpha-induced protein 8-like 3 (TNFAIP8L3)	0.714(0.0342)	0.559(0.0113)	0.783
LOC654103	similar to solute carrier family 25, member 37 (LOC654103)	1.656(0.0237)	1.267(0.1425)	0.765
HSD17B2	hydroxysteroid (17-beta) dehydrogenase 2 (HSD17B2)	3.470(0.0273)	2.654(0.0370)	0.764
PFKL	phosphofructokinase, liver (PFKL), transcript variant 2	1.566(0.0480)	1.188(0.4919)	0.758
P2RX4	purinergic receptor P2X, ligand-gated ion channel, 4 (P2RX4)	1.601(0.0239)	1.208(0.2289)	0.754
SLC16A5	solute carrier family 16, member 5 (monocarboxylic acid transporter 6) (SLC16A5)	1.711(0.0299)	1.289(0.1066)	0.753
STX11	syntaxin 11 (STX11)	1.825(0.0201)	1.372(0.0375)	0.751
AK3L1	adenylate kinase 3-like 1 (AK3L1), nuclear gene encoding mitochondrial protein, transcript variant 7	1.980(0.0045)	1.478(0.9522)	0.746
ECE1	endothelin converting enzyme 1 (ECE1)	1.597(0.0368)	1.193(0.1296)	0.746
GPX7	glutathione peroxidase 7 (GPX7)	1.794(0.0069)	1.336(0.0984)	0.744
EGLN1	egl nine homolog 1 (C. elegans) (EGLN1)	1.504(0.0057)	1.114(0.2252)	0.740
SC65	synaptonemal complex protein SC65 (SC65)	1.515(0.0182)	1.110(0.4886)	0.733
TMEM158	transmembrane protein 158 (TMEM158)	2.285(0.0186)	1.654(0.2429)	0.723
LOC646821	similar to beta-actin (LOC646821)	1.652(0.0156)	1.192(0.1170)	0.721
LOC286016	triosephosphate isomerase 1 pseudogene (LOC286016), non-coding RNA.	1.937(0.0029)	1.376(0.0251)	0.710
PFKP	phosphofructokinase, platelet (PFKP)	1.730(6.682e-05)	1.227(0.2618)	0.709
PGM1	phosphoglucomutase 1 (PGM1)	2.141(0.0153)	1.516(0.1655)	0.708
RCN3	reticulocalbin 3, EF-hand calcium binding domain (RCN3)	1.956(0.0200)	1.383(0.0037)	0.707
SPAG4	sperm associated antigen 4 (SPAG4)	2.219(0.0012)	1.546(0.0183)	0.696
ADORA2A	adenosine A2a receptor (ADORA2A)	1.885(0.0011)	1.293(0.1982)	0.685
PFKFB4	6-phosphofructo-2-kinase/fructose-2,6-biphosphatase 4 (PFKFB4)	1.682(0.0008)	1.152(0.0305)	0.685
SLC25A37	solute carrier family 25, member 37 (SLC25A37), nuclear gene encoding mitochondrial protein	1.559(0.0082)	1.067(0.7086)	0.684
STC2	stanniocalcin 2 (STC2)	3.640(0.0049)	2.459(0.0765)	0.675

### Validation of array results

**Fig 3:**
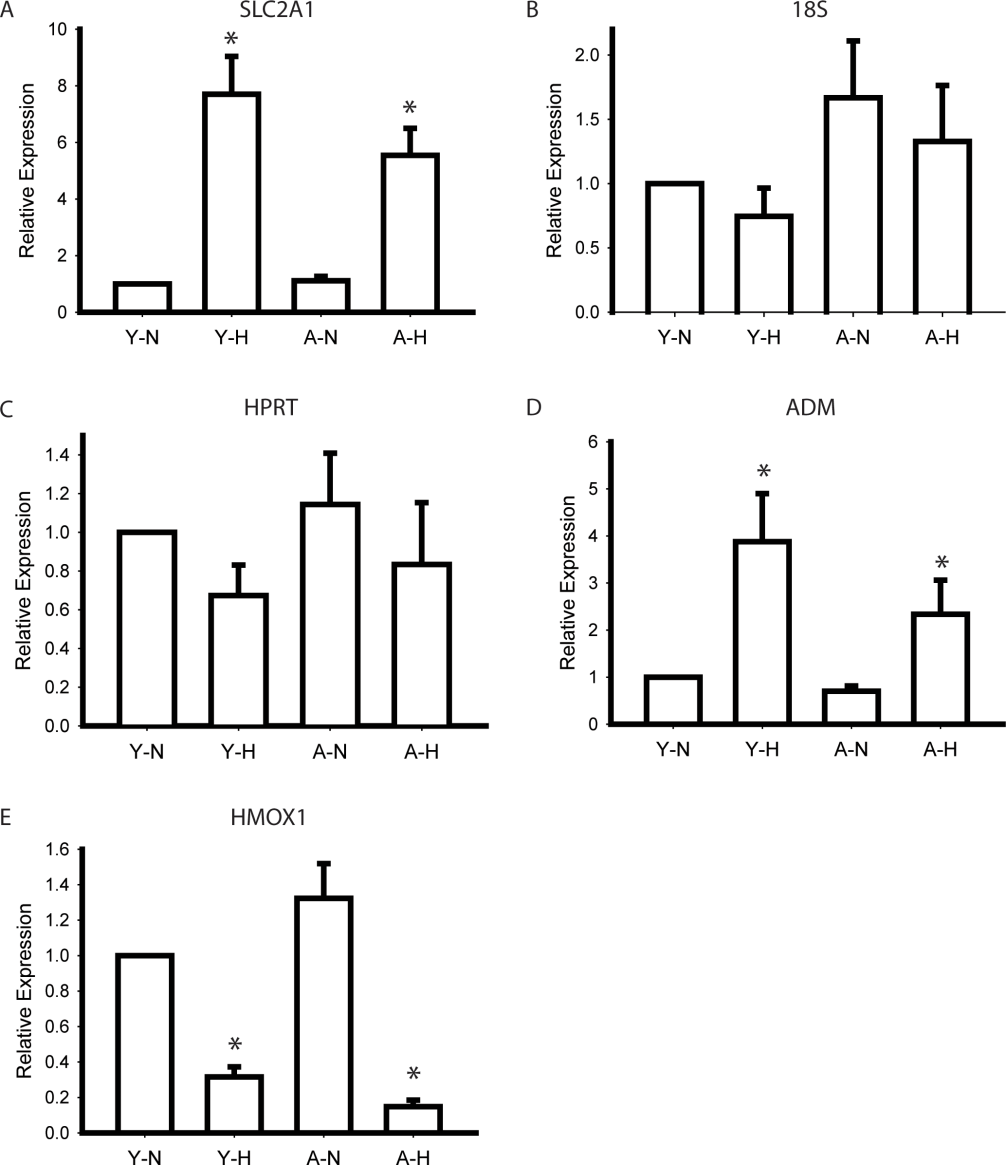
Quantitative PCR for control genes. (A) SLC2A1 was significantly up-regulated by hypoxia. No significant difference was found in 18S (B) and HPRT (C) expression. (D) ADM was significantly upregulated and (E) HMOX1 was downregulated by hypoxia which was consistent with microarray results. N = 6 * P < .05 compared to normoxia cells of the same passage. Y: young; A: aged; N: normoxia; H: hypoxia.

To validate our microarray results from 3 CD34^+^ EPCs clones, we performed Q-PCR analysis by using 6 additional EPCs clones. SLC2A1 was the gene associated with the highest fold changes in response to hypoxia in young and aged CD34^+^ EPCs (Table 1). Our results indicated that SLC2A1 expression was affected by factors additional to aging (Fig 3A). Our real-time PCR and microarray results were consistent, indicating a > 4-fold increase in SLC2A1 expression in response to hypoxia. When we evaluated the expression of genes unaffected by aging and hypoxia, including 18S and HPRT, our Q-PCR results indicated nonsignificant differences in gene expression between the different conditions (Fig 3B and 3C). We also evaluated genes that were affected by hypoxia irrespective of the passage number of cells, including ADM, which was upregulated by hypoxia, and HMOX1, which was downregulated by hypoxia (Fig 3D and 3E). Our results on the changes in ADM and HMOX1 expression were consistent with our microarray results, indicating that our microarray results are reliable.

**Fig 4:**
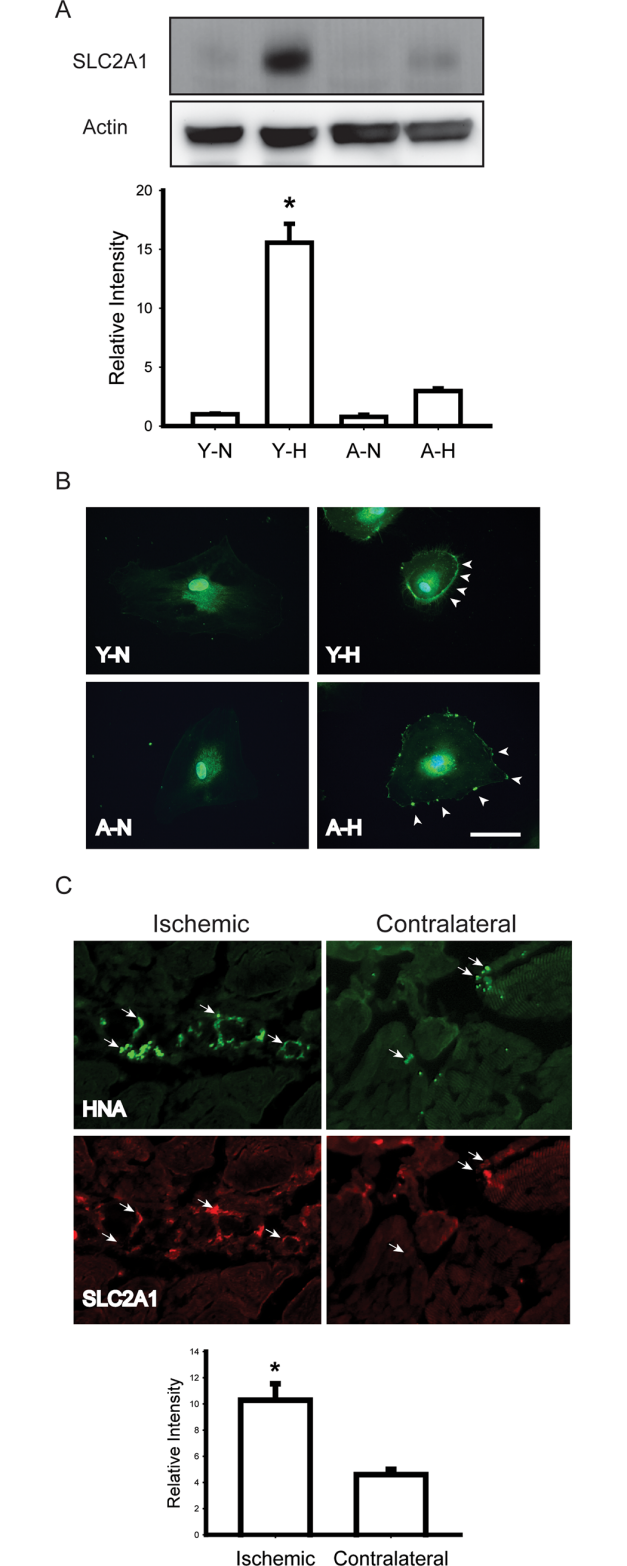
Validation of SLC2A1 expression in response to hypoxia. (A) Western blots showing a significant rise of the SLC2A1 protein level in young EPCs. Representative Western blots and normalized quantification of band density were shown. * p < .05 compared to young CD34^+^ EPCs in normoxia, N = 3, Bars represent mean ± SEM. (B) Hypoxia-induced membrane translocation of SLC2A1 (Bar = 10mm). (C) EPCs (labeled with HNA) injected into ischemic hindlimb (Left) expressed more SLC2A1 that EPCs injected into the contralateral tissue (Right). Y: young; A: aged; N: normoxia; H: hypoxia; HNA: human nuclear antigen.

We also detected the protein expression level of SLC2A1 with Western blot (Fig 4A). In line with the microarray and Q-PCR results, hypoxia also induced significant rise of SLC2A1 proteins in young CD34^+^ EPCs. The rise in mRNA in aged CD34^+^ EPCs only gave rise to a moderate increase of proteins indicating the involvement translation or protein stability in aged CD34^+^ EPCs. Immunofluorescence staining on EPCs revealed membrane translocation induced by hypoxia (Fig 4B). To test whether the same hypoxia responses happened in vivo, we created hindlimb ischemia in mice by ligating the right femoral artery and vein. CD34^+^ EPCs were then injected into both thighs and calfs for 48 hours. The expression of SLC2A1 in injected CD34^+^ EPCs was detected (Fig 4C). The locations of CD34^+^ EPCs were labeled with human nuclear antigen (HNA) antibodies (Fig 4C HNA). The HNA signals colocalized with SLC2A1 signaling in the ischemic side (Left) but not the contralateral side (Right). We quantified the expression intensity of SLC2A1 within HNA positive regions with Metamorph software and the intensity was significantly higher on the ischemic side than the contralateral side. Our results demonstrated that, consistent with our array data, the expression level of SLC2A1 was elevated in response to hypoxia both in vitro and in vivo.

### Gene set enrichment analysis

To further evaluate the relevance of differential gene expression, we performed gene function analysis, grouping the genes according to gene ontology terms. The gene ontology (GO) group “response to oxygen level,” which contains the genes HOMX1, BNIP3, and ALDOC, was associated with greatest changes in response to hypoxia, followed by “cardiovascular system development.” Genes belonging to a common gene set, such as HOMX1, were regulated by hypoxia in both young and aged groups. Therefore, to evaluate the differing responses to hypoxia in young and aged CD34^+^ EPCs, we eliminated the common genes and performed GSEA of genes in the “young only” (Table 2) and “aged only” sets (Table 3). Comparing Tables 2 and 3, we observed that genes involved in response to oxygen levels, such as BNIP3, were upregulated in young EPCs only. As shown in Table 2, the GO terms “fructose metabolic process” and “glycolysis” indicated a shift in metabolism in young CD34^+^ EPCs in hypoxic conditions. We did not observe such changes in gene expression in aged CD34^+^ EPCs.

**Table 2:** GSEA results in differentially expressed genes (young only).

GO terms	Gene lists	p-value
GO:0070482 response to oxygen levels	BNIP3,ALDOC,STC2,NOS3,TGFBR3,ECE1,TFRC,SERPINE1,PGF,PLOD1,EGLN1	1.60E-08
GO:0006000 fructose metabolic process	PFKP,PFKFB4,ALDOC,PFKL	1.10E-06
GO:0030388 fructose 1,6-bisphosphate metabolic process	PFKP,ALDOC,PFKL	2.70E-06
GO:0008285 negative regulation of cell proliferation	ADORA2A,NOX4,NOS3,TGFBR3,JUN,HSPA1B,BTG2,ID2,NACC2,SESN1	9.70E-05
GO:0006096 glycolysis	ALDOC,PFKP,PFKL,PFKFB4	0.0001
GO:0003100 regulation of systemic arterial blood pressure by endothelin	NOS3,ECE1	0.0001
GO:0090083 regulation of inclusion body assembly	HSPA1B,SNCAIP	0.0001
GO:0008443 phosphofructokinase activity	PFKP,PFKFB4,PFKL	2.60E-06
GO:0070095 fructose-6-phosphate binding	PFKP,PFKL	0.0001
GO:0015355 secondary active monocarboxylate transmembrane transporter activity	SLC16A5,SLC16A3	0.0001
GO:0005515 protein binding	LDB2,P2RX4,RASGRP3,ADORA2A,SQSTM1,PFKFB4,STC2,NOS3,TGFBR3,DPYSL3,TFRC,PGF,CXCL16,FLRT2,SYNCRIP,SRPX2,STX11,EFEMP2,SERPINE1,JUN,HSPA1B,COL4A2,ALDOC,NQO1,PFKL,COL5A1,PTPRB,LMO2,LOX,DUSP1,ECE1,BTG2,TAGLN,ID2,PLOD1,BNIP3,MALL,KANK1,TGFBI,MTHFD1L,NACC2,RCN3,NOTCH4,AIF1L,EGLN1,FAM124B,SNCAIP	0.0002

**Table 3:** GSEA results in differentially expressed genes (old only).

GO terms	Gene lists	p-value
GO:0030204 chondroitin sulfate metabolic process	VCAN,SDC4,CHST15	5.40E-05
GO:0001659 temperature homeostasis	GPX1,APLN	0.0008
GO:0009617 response to bacterium	ABCA1,GPX1,SELE,VLDLR	0.001
GO:0001682 tRNA 5'-leader removal	POP1	0.0013
GO:0016078 tRNA catabolic process	POP1	0.0013
GO:0038027 apolipoprotein A-I-mediated signaling pathway	ABCA1	0.0013
GO:0050859 negative regulation of B cell receptor signaling pathway	LPXN	0.0013
GO:0042311 vasodilation	GPX1,APLN	0.0016
GO:0034185 apolipoprotein binding	ABCA1,VLDLR	0.0001
GO:0000171 ribonuclease MRP activity	POP1	0.0012
GO:0030226 apolipoprotein receptor activity	ABCA1	0.0012
GO:0031704 apelin receptor binding	APLN	0.0012
GO:0016893 endonuclease activity, active with either ribo- or deoxyribonucleic acids and producing 5'-phosphomonoesters	POP1,DNASE1L3	0.0015

## Discussion

In this study, we profiled hypoxia-induced gene expression in young and aged CD34^+^ EPCs by using microarray analysis, and confirmed our results by using real-time PCR, Western blotting, and animal model. Our results indicate the effects of senescence on EPCs responses to hypoxia. Differing CD34^+^ EPCs responses might underlie increased risk of ischemic diseases associated with age. With the potential applications of EPCs in cardiovascular and other diseases, our study provides insight on the impact of ex vivo expansion might have on EPCs.

The concentration of circulating EPCs has been inversely correlated with the health of cardiovascular system [32]. Other than the change in circulating EPCs numbers, changes in EPCs properties including migratory and proliferative activities [33] and telomere length [34] were also reported in patients with cardiovascular diseases. Kushner et al. demonstrated that EPCs telomere length declines with age even in healthy men [35]. For our study, it would have been ideal to obtain EPCs from young and aged subjects to represent the different status of EPCs. However, premature senescence of EPCs and reduced telomere length were also observed in healthy young adults with family history of cardiovascular diseases [36] and the physiological age may not represent the true health status of an individual. Ex vivo expansion of EPCs was shown to induce cell-cycle arrest with hallmarks of cellular senescence such as β-gal expression, telomere shortening [27]. Cellular senescence is driven by tumor suppressor pathway. The phosphorylation level of the key molecular of the pathway, p53, was increased and expressions of pRb and p21were up-regulated in late EPCs passages as expected [27]. We understand that replicative senescence model may not totally reflect the differences between young and age subjects. Without the full understanding of the genetic background of our volunteer donors, we choose this model to reduce the effects of individual difference. Collecting clinical samples from subjects at different ages will be a critical validation of our results in this study.

Hypoxia upregulates the expression of angiogenic growth factors, attracts progenitor cells expressing angiogenic growth factor receptors, and increases the number of circulating angiogenic cells (CACs)[37]. In addition to angiogenic growth factor receptors, CACs express progenitor cell markers such as CD34 and Sca1 [23]. Studies have shown that ischemic-induced mobilization of CACs is impaired in older mice [38]. Changes in the surface markers of CACs could potentially be used as biomarkers of ischemic hypoxia. Martin-Rendon et al (2007) conducted hypoxic gene profiling of umbilical cord blood and bone marrow-derived stem cells and observed differences in the hypoxia-induced expression of 200 genes [39]. However, the hypoxic gene profile of EPCs, the major component of CACs, is not well described. In this study, we established the hypoxic gene profiles of young and aged CD34^+^ EPCs, and identified factors that could potentially be used as biomarkers of aging-related diseases. We compared CD34^+^ EPCs in the hypoxia (1% oxygen) conditions with atmospheric oxygen (20%) in the current study, it would be of great interest to compare them with a more physiological oxygen environment (such as 5%) in the future.

EPCs might contribute to neovascularization through direct incorporation into regenerating vasculature [24], as well as through the indirect production of proangiogenic cytokines [40]. Kalka et al first used culture-expanded EPCs as a single therapeutic agent for hind limb ischemia [41]. Subsequent studies transplanted human EPCs into animals to improve outcome from various cardiovascular diseases models [41,42]. Clinical trials have aimed to replicate the effects observed in animal models of human ischemic diseases [37]. Preliminary results from such trials indicated that EPC therapy is safe and feasible. However, the number of available EPCs might represent a limiting factor for successful application in humans. Many protocols tackle this challenge with ex vivo expansion of EPCs and the replicative senescence of EPCs in culture becomes a critical issue for successful clinical trials. By profiling EPCs and their replicative senescence counterparts, our study provided information to identify the key factors affecting the proliferative ability of EPCs. From our GSEA results, we found that young CD34^+^ EPCs switched their metabolic pathway and turned on their hypoxia-responsive genes in response to the change in the oxygen environment. However, aged CD34^+^ EPCs did not successfully turn on those genes, which might lead to subsequence EPCs dysfunction and cell death.

Impaired angiogenesis and vascular remodeling in age-associated ischemic hypoxia are associated with progressive impairment of ischemic-induced hypoxia-inducible factor 1 (HIF-1), which activates the transcription of hundreds of target genes in response to reduced oxygen availability [38,43,44]. It is well established that HIF-1 is the key regulator of oxygen homeostasis, and activates target genes that contain hypoxic response elements [45]. Under atmospheric oxygen conditions, the HIF-1α subunit is subjected to oxygen-dependent asparaginyl hydroxylation and prolyl hydroxylation, leading to loss of transcriptional activity and proteasomal degradation [46]. We observed no transcriptional change of HIF-1 between different oxygen environments in neither young nor aged cells. However, the expression of a major prolyl hydroxylation enzyme, egl nine homolog 1 (EGLN1, also known as prolyl hydroxylase domain-containing protein 2 (PHD2)), was regulated by hypoxia only in young EPCs indicating a lack of HIF-1 regulation in aged EPCs. HIF-1 modulates angiogenesis and vascular remodeling through the regulation of angiogenic growth factors, including VEGF and placental growth factor [47–49]. We detected hypoxia induction of placental growth factor only in young CD34^+^ EPC while VEGF was upregulated in both young and aged CD34^+^ EPCs; hinted two different regulatory pathways exist. These angiogenic growth factors activate resident endothelial cells and vascular pericytes to participate in vascular remodeling and mobilize and recruit EPCs, mesenchymal stem cells, hematopoietic stem cells, and myeloid cells [25,38,50,51]. Our result is consistent with previous research that aging is associated with the progressive downregulation of ischemic-induced HIF-1α protein and angiogenic growth factors expression [38].

Mitochondria are organelles with high oxygen demand to maintain normal functions in the respiratory chain. They are considered major contributors to the aging process because of the generation of ROS during respiration. Under hypoxic conditions, mitochondrial respiration switches to glycolysis [52]. HIF-1 plays a major role in increasing glycolysis by upregulating the expression of glycolytic enzymes and glucose transporters [53]. One of these genes is solute carrier family 2 (facilitated glucose transporter) member 1(SLC2A1), otherwise known as glucose transporter 1, a major transporter of glucose across plasma membranes. SLC2A1 is critical to a cell’s energy supply in conditions of insufficient oxygen and enables cellular respiration in hypoxic conditions when the cell increasingly relies on glycolysis [54]. We observed upregulation of these genes only in young CD34^+^ EPCs, which further indicates the dysregulation of HIF-1 activity in response to hypoxia in aged CD34^+^ EPCs.

Mitochondria also play a cell death-related role. Defective mitochondria lose its functions and, if unregulated, ultimately release proapoptotic factors, causing cell death. Therefore, the cell death-related effects of mitochondria must be strictly controlled. Organelle quality control promotes the sequestration, sorting, and elimination of defective mitochondria through fusion, fission, and autophagy [55–57]. Bcl-2 nineteen kilodalton-protein interacting protein 3 (BNIP3) is the key regulator of apoptosis, necrosis, and autophagy in response to hypoxia. Studies have extensively investigated the role of BNIP3 in response to heart disease [58], and cerebral ischemia [59]. Daido et al (2004) first described the role of BNIP3 in autophagy in malignant glioma [60]. Subsequent studies identified BNIP3-induced autophagy in other cell types in response to stress [61–66]. In previous studies, hypoxia upregulated BINP3 expression in neonatal cardiomyocytes and adult ventricular myocytes [67–69]. In animal studies, BNIP3 expression was induced by ischemia and upregulated in chronic heart failure or intermittent hypoxic challenge [70–72]. In Jurasz et al (2011), BNIP3 was upregulated by 24–48 hours of hypoxia in endothelial cells [73]. However, the role of BNIP3 in aging in endothelial cells or EPCs is not well understood. In this study, we identified that hypoxia upregulates BNIP3 gene expression in young, but not aged, CD34^+^ EPCs, indicating a potential role of BNIP3 in aging-related cardiovascular diseases.

In summary, our study results indicate the mechanisms underlying and consequences of CD34^+^ EPCs aging, and the CD34^+^ EPCs response to hypoxia. Our results could facilitate future investigation of the role of mitochondria in CD34^+^ EPCs aging and associated diseases, and the identification of markers for the prediction and diagnosis of aging-related diseases.

## Supporting information

S1 Table All genes changed by hypoxia in young but not old EPCs. (DOCX)

S2 Table All genes changed by hypoxia in old but not young EPCs. (DOCX)

S3 Table All genes changed by hypoxia in both young and old EPCs. (DOCX)
