# Supplementary material for: Genetic profiling of young and aged endothelial progenitor cells in hypoxia
Source: PLoS One. 2018 Apr 30;13(4):e0196572. doi: 10.1371/journal.pone.0196572 (PMC5927426; doi:10.1371/journal.pone.0196572)
Supplement: S1 Table — (DOCX) [file pone.0196572.s001.docx]

**S1 Table All genes changed by hypoxia in young but not old EPCs.**

| **Gene Symbol** | **Gene Description** | **fold change (p-value), Young (hy/normal)** | **fold change (p-value), Old (hy/normal)** | **fold change (Old/Young)** |
| --- | --- | --- | --- | --- |
| SLCO2A1 | solute carrier organic anion transporter family, member 2A1 (SLCO2A1) | 0.276(0.0284) | 0.831(0.1742) | 3.002 |
| ID2 | inhibitor of DNA binding 2, dominant negative helix-loop-helix protein (ID2) | 0.645(0.0145) | 1.358(0.2341) | 2.105 |
| LHFPL2 | lipoma HMGIC fusion partner-like 2 (LHFPL2) | 0.615(0.0267) | 1.104(0.4028) | 1.795 |
| NQO1 | NAD(P)H dehydrogenase, quinone 1 (NQO1), transcript variant 1 | 0.242(0.0011) | 0.409(0.1437) | 1.691 |
| LGALS9 | lectin, galactoside-binding, soluble, 9 (LGALS9), transcript variant 1 | 0.614(0.0352) | 1.017(0.7327) | 1.654 |
| HSPA1B | heat shock 70kDa protein 1B (HSPA1B) | 0.609(0.0140) | 0.929(0.7170) | 1.523 |
| MALL | mal, T-cell differentiation protein-like (MALL) | 0.611(0.0476) | 0.919(0.7652) | 1.504 |
| LOC100129759 | similar to PNAS-117 (LOC100129759) | 0.657(0.0136) | 0.988(0.9303) | 1.502 |
| FLRT2 | fibronectin leucine rich transmembrane protein 2 (FLRT2) | 0.418(0.0151) | 0.628(0.1131) | 1.5 |
| SESN1 | sestrin 1 (SESN1) | 0.644(0.0089) | 0.964(0.3835) | 1.497 |
| COL4A2 | collagen, type IV, alpha 2 (COL4A2) | 2.105(0.0036) | 1.400(0.0208) | 0.665 |
| SLC16A3 | solute carrier family 16, member 3 (monocarboxylic acid transporter 4) (SLC16A3), transcript variant 2 | 1.798(0.0222) | 1.171(0.6710) | 0.651 |
| PGF | placental growth factor (PGF) | 1.767(0.0401) | 1.146(0.6088) | 0.648 |
| EFEMP2 | EGF-containing fibulin-like extracellular matrix protein 2 (EFEMP2) | 2.009(0.0036) | 1.293(0.1873) | 0.643 |
| GGT3 | misc_RNA (GGT3) | 2.057(0.0014) | 1.320(0.0960) | 0.641 |
| NOX4 | NADPH oxidase 4 (NOX4), transcript variant 1 | 1.960(0.0484) | 1.253(0.2460) | 0.639 |
| BNIP3 | BCL2/adenovirus E1B 19kDa interacting protein 3 (BNIP3), nuclear gene encoding mitochondrial protein | 1.843(0.0201) | 1.164(0.3878) | 0.631 |
| ECGF1 | endothelial cell growth factor 1 (platelet-derived) (ECGF1) | 2.188(0.0204) | 1.356(0.0338) | 0.62 |
| AK1 | adenylate kinase 1 (AK1) | 1.860(0.0224) | 1.140(0.5647) | 0.613 |
| ALDOC | aldolase C, fructose-bisphosphate (ALDOC) | 2.386(0.0004) | 1.464(0.0244) | 0.613 |
| HYI | hydroxypyruvate isomerase homolog (E. coli) (HYI) | 1.861(0.0243) | 1.135(0.1143) | 0.61 |
| AXUD1 | AXIN1 up-regulated 1 (AXUD1) | 1.913(0.0257) | 1.144(0.5125) | 0.598 |
| SERPINE1 | serpin peptidase inhibitor, clade E (nexin, plasminogen activator inhibitor type 1), member 1 (SERPINE1) | 2.290(0.0327) | 1.338(0.2615) | 0.584 |
| TGFBI | transforming growth factor, beta-induced, 68kDa (TGFBI) | 3.258(0.0002) | 1.897(0.2238) | 0.582 |
| SRPX2 | sushi-repeat-containing protein, X-linked 2 (SRPX2) | 1.767(0.0120) | 1.028(0.9488) | 0.581 |
| DUSP1 | dual specificity phosphatase 1 (DUSP1) | 2.387(0.0062) | 1.387(0.0918) | 0.58 |
| TAGLN | transgelin (TAGLN), transcript variant 1 | 4.006(0.0024) | 2.088(0.3950) | 0.521 |
| SNCAIP | synuclein, alpha interacting protein (SNCAIP) | 2.080(0.0291) | 1.078(0.0217) | 0.518 |
| ADSSL1 | adenylosuccinate synthase like 1 (ADSSL1), transcript variant 2 | 1.818(7.199e-06) | 0.935(0.3896) | 0.514 |
| C13ORF15 | chromosome 13 open reading frame 15 (C13orf15) | 2.234(0.0185) | 1.082(0.7791) | 0.484 |
| C17ORF79 | chromosome 17 open reading frame 79 (C17orf79) | 0.641(0.0278) | 0.900(0.1720) | 1.404 |
| BTG2 | BTG family, member 2 (BTG2) | 0.660(0.0292) | 0.921(0.6923) | 1.395 |
| LFNG | LFNG O-fucosylpeptide 3-beta-N-acetylglucosaminyltransferase (LFNG), transcript variant 1 | 0.604(0.0355) | 0.842(0.0621) | 1.393 |
| TGFBR3 | transforming growth factor, beta receptor III (TGFBR3) | 0.621(0.0331) | 0.854(0.0976) | 1.373 |
| CXCL16 | chemokine (C-X-C motif) ligand 16 (CXCL16) | 0.580(0.0463) | 0.794(0.0575) | 1.368 |
| AIF1L | allograft inflammatory factor 1-like (AIF1L), transcript variant 1 | 0.606(0.0046) | 0.829(0.6684) | 1.367 |
| SYNCRIP | synaptotagmin binding, cytoplasmic RNA interacting protein (SYNCRIP) | 0.652(0.0351) | 0.856(0.0291) | 1.313 |
| LOC644033 | similar to similar to RPL23AP7 protein (LOC644033) | 0.639(0.0455) | 0.838(0.2685) | 1.31 |
| PRRG1 | proline rich Gla (G-carboxyglutamic acid) 1 (PRRG1) | 0.650(0.0101) | 0.839(0.3646) | 1.291 |
| AKR1B1 | aldo-keto reductase family 1, member B1 (aldose reductase) (AKR1B1) | 0.631(0.0211) | 0.785(0.1894) | 1.243 |
| SEC11C | SEC11 homolog C (S. cerevisiae) (SEC11C) | 0.632(0.0007) | 0.775(0.1333) | 1.225 |
| NOS3 | nitric oxide synthase 3 (endothelial cell) (NOS3) | 0.589(0.0052) | 0.706(0.2137) | 1.198 |
| SQSTM1 | sequestosome 1 (SQSTM1) | 0.586(0.0151) | 0.666(0.0765) | 1.136 |
| FAM124B | family with sequence similarity 124B (FAM124B), transcript variant 2 | 0.606(0.0049) | 0.674(0.0432) | 1.112 |
| TFRC | transferrin receptor (p90, CD71) (TFRC) | 0.462(0.0143) | 0.499(0.1856) | 1.078 |
| LYVE1 | lymphatic vessel endothelial hyaluronan receptor 1 (LYVE1) | 0.318(0.0434) | 0.343(0.1687) | 1.078 |
| LMO2 | LIM domain only 2 (rhombotin-like 1) (LMO2) | 0.615(0.0102) | 0.663(0.0519) | 1.077 |
| LOX | lysyl oxidase (LOX) | 2.711(0.0071) | 2.898(0.1194) | 1.068 |
| ZNF323 | zinc finger protein 323 (ZNF323), transcript variant 1 | 0.629(0.0008) | 0.666(0.0622) | 1.058 |
| LDB2 | LIM domain binding 2 (LDB2) | 0.479(0.0168) | 0.495(0.0753) | 1.035 |
| ODC1 | ornithine decarboxylase 1 (ODC1) | 0.622(0.0475) | 0.609(0.0681) | 0.979 |
| ERO1L | ERO1-like (S. cerevisiae) (ERO1L) | 1.848(0.0068) | 1.793(0.1016) | 0.969 |
| RASGRP3 | RAS guanyl releasing protein 3 (calcium and DAG-regulated) (RASGRP3) | 0.652(0.0344) | 0.611(0.2326) | 0.938 |
| COL5A1 | collagen, type V, alpha 1 (COL5A1) | 1.812(0.0216) | 1.650(0.1530) | 0.91 |
| KANK1 | KN motif and ankyrin repeat domains 1 (KANK1), transcript variant 1 | 1.687(0.0322) | 1.520(0.0564) | 0.9 |
| LOC441763 | hypothetical LOC441763 (LOC441763) | 1.619(0.0133) | 1.445(0.2423) | 0.892 |
| TMEM91 | transmembrane protein 91 (TMEM91) | 1.563(0.0125) | 1.366(0.0652) | 0.873 |
| FER1L4 | fer-1-like 4 (C. elegans) (FER1L4) on chromosome 20. | 2.108(0.0313) | 1.831(0.0569) | 0.868 |
| NACC2 | NACC family member 2, BEN and BTB (POZ) domain containing (NACC2) | 0.623(0.0009) | 0.537(0.0593) | 0.862 |
| CRELD1 | cysteine-rich with EGF-like domains 1 (CRELD1), transcript variant 3 | 1.602(0.0194) | 1.364(0.1157) | 0.851 |
| MTHFD1L | methylenetetrahydrofolate dehydrogenase (NADP+ dependent) 1-like (MTHFD1L) | 1.606(0.0243) | 1.361(0.2833) | 0.847 |
| LOC644237 | misc_RNA (LOC644237) | 1.674(0.0033) | 1.405(0.0056) | 0.839 |
| PTPRB | protein tyrosine phosphatase, receptor type, B (PTPRB) | 1.682(0.0373) | 1.402(0.0417) | 0.833 |
| PLOD1 | procollagen-lysine 1, 2-oxoglutarate 5-dioxygenase 1 (PLOD1) | 1.567(0.0295) | 1.290(0.5383) | 0.823 |
| JUN | jun oncogene (JUN) | 1.569(0.0445) | 1.289(0.1569) | 0.821 |
| DPYSL3 | dihydropyrimidinase-like 3 (DPYSL3) | 1.625(0.0164) | 1.322(0.0703) | 0.813 |
| FBLN7 | fibulin 7 (FBLN7) | 1.718(0.0257) | 1.388(0.1189) | 0.807 |
| GAPDHL6 | glyceraldehyde-3-phosphate dehydrogenase-like 6 (GAPDHL6) | 1.707(0.0111) | 1.359(0.0045) | 0.796 |
| NOTCH4 | Notch homolog 4 (Drosophila) (NOTCH4) | 1.517(0.0099) | 1.204(0.2134) | 0.793 |
| LOC732007 | similar to Phosphoglycerate mutase 1 (Phosphoglycerate mutase isozyme B) (PGAM-B) (BPG-dependent PGAM 1) (LOC732007) | 1.516(0.0379) | 1.197(0.1961) | 0.789 |
| LOC654103 | similar to solute carrier family 25, member 37 (LOC654103) | 1.656(0.0237) | 1.267(0.1425) | 0.765 |
| PFKL | phosphofructokinase, liver (PFKL), transcript variant 2 | 1.566(0.0480) | 1.188(0.4919) | 0.758 |
| P2RX4 | purinergic receptor P2X, ligand-gated ion channel, 4 (P2RX4) | 1.601(0.0239) | 1.208(0.2289) | 0.754 |
| SLC16A5 | solute carrier family 16, member 5 (monocarboxylic acid transporter 6) (SLC16A5) | 1.711(0.0299) | 1.289(0.1066) | 0.753 |
| STX11 | syntaxin 11 (STX11) | 1.825(0.0201) | 1.372(0.0375) | 0.751 |
| AK3L1 | adenylate kinase 3-like 1 (AK3L1), nuclear gene encoding mitochondrial protein, transcript variant 7 | 1.980(0.0045) | 1.478(0.9522) | 0.746 |
| ECE1 | endothelin converting enzyme 1 (ECE1) | 1.597(0.0368) | 1.193(0.1296) | 0.746 |
| GPX7 | glutathione peroxidase 7 (GPX7) | 1.794(0.0069) | 1.336(0.0984) | 0.744 |
| EGLN1 | egl nine homolog 1 (C. elegans) (EGLN1) | 1.504(0.0057) | 1.114(0.2252) | 0.74 |
| SC65 | synaptonemal complex protein SC65 (SC65) | 1.515(0.0182) | 1.110(0.4886) | 0.733 |
| TMEM158 | transmembrane protein 158 (TMEM158) | 2.285(0.0186) | 1.654(0.2429) | 0.723 |
| LOC646821 | similar to beta-actin (LOC646821) | 1.652(0.0156) | 1.192(0.1170) | 0.721 |
| LOC286016 | triosephosphate isomerase 1 pseudogene (LOC286016), non-coding RNA. | 1.937(0.0029) | 1.376(0.0251) | 0.71 |
| PFKP | phosphofructokinase, platelet (PFKP) | 1.730(6.682e-05) | 1.227(0.2618) | 0.709 |
| PGM1 | phosphoglucomutase 1 (PGM1) | 2.141(0.0153) | 1.516(0.1655) | 0.708 |
| RCN3 | reticulocalbin 3, EF-hand calcium binding domain (RCN3) | 1.956(0.0200) | 1.383(0.0037) | 0.707 |
| ADORA2A | adenosine A2a receptor (ADORA2A) | 1.885(0.0011) | 1.293(0.1982) | 0.685 |
| PFKFB4 | 6-phosphofructo-2-kinase/fructose-2,6-biphosphatase 4 (PFKFB4) | 1.682(0.0008) | 1.152(0.0305) | 0.685 |
| SLC25A37 | solute carrier family 25, member 37 (SLC25A37), nuclear gene encoding mitochondrial protein | 1.559(0.0082) | 1.067(0.7086) | 0.684 |
| STC2 | stanniocalcin 2 (STC2) | 3.640(0.0049) | 2.459(0.0765) | 0.675 |
