# Supplementary material for: Genetic profiling of young and aged endothelial progenitor cells in hypoxia
Source: PLoS One. 2018 Apr 30;13(4):e0196572. doi: 10.1371/journal.pone.0196572 (PMC5927426; doi:10.1371/journal.pone.0196572)
Supplement: S2 Table — (DOCX) [file pone.0196572.s002.docx]

**S2 Table All genes changed by hypoxia in old but not young EPCs.**

| **Gene Symbol** | **Gene Description** | **fold change (p-value), Young (hy/normal)** | **fold change (p-value), Old (hy/normal)** | **fold change (Old/Young)** |
| --- | --- | --- | --- | --- |
| FAM107A | family with sequence similarity 107, member A (FAM107A), transcript variant 2 | 1.043(0.9038) | 2.178(0.0160) | 2.088 |
| DNASE1L3 | deoxyribonuclease I-like 3 (DNASE1L3) | 0.870(0.4691) | 1.651(0.0115) | 1.896 |
| ABCA1 | ATP-binding cassette, sub-family A (ABC1), member 1 (ABCA1) | 0.876(0.1384) | 1.543(0.0110) | 1.761 |
| DDIT4 | DNA-damage-inducible transcript 4 (DDIT4) | 1.346(0.0430) | 2.237(0.0228) | 1.661 |
| SNORD13 | small nucleolar RNA, C/D box 13 (SNORD13), small nucleolar RNA. | 1.147(0.6815) | 1.771(0.0392) | 1.544 |
| ITM2B | integral membrane protein 2B (ITM2B) | 1.031(0.7240) | 1.581(0.0040) | 1.533 |
| LPXN | leupaxin (LPXN) | 0.809(0.0559) | 0.498(0.0241) | 0.615 |
| SELE | selectin E (endothelial adhesion molecule 1) (SELE) | 0.979(0.7610) | 0.590(0.0314) | 0.603 |
| TFPI2 | tissue factor pathway inhibitor 2 (TFPI2) | 1.097(0.6249) | 0.633(0.0156) | 0.577 |
| MSMP | microseminoprotein, prostate associated (MSMP) | 1.135(0.5134) | 0.604(0.0043) | 0.532 |
| LOC646723 | similar to Keratin, type I cytoskeletal 18 (Cytokeratin-18) (CK-18) (Keratin-18) (K18) (LOC646723) | 0.919(0.6277) | 0.455(0.0495) | 0.495 |
| ERRFI1 | ERBB receptor feedback inhibitor 1 (ERRFI1) | 2.524(0.1770) | 3.665(0.0200) | 1.452 |
| PLIN5 | perilipin 5 (PLIN5) | 1.100(0.6499) | 1.527(0.0385) | 1.388 |
| LOC375295 | hypothetical gene supported by BC013438 (LOC375295) | 1.474(0.1006) | 1.995(0.0085) | 1.353 |
| DUXAP3 | double homeobox A pseudogene 3 (DUXAP3) on chromosome 10. | 1.206(0.2747) | 1.633(0.0103) | 1.353 |
| LOC441087 | hypothetical gene supported by AK125735 (LOC441087) | 1.188(0.1521) | 1.539(0.0013) | 1.294 |
| GPR126 | G protein-coupled receptor 126 (GPR126), transcript variant a2 | 1.468(0.4152) | 1.879(0.0078) | 1.279 |
| VCAN | versican (VCAN) | 1.948(0.0784) | 2.429(0.0028) | 1.246 |
| CHD1L | chromodomain helicase DNA binding protein 1-like (CHD1L) | 1.254(0.1101) | 1.549(0.0440) | 1.235 |
| SDC4 | syndecan 4 (SDC4) | 1.315(0.2486) | 1.622(0.0150) | 1.233 |
| LOC643031 | similar to NADH dehydrogenase subunit 5 (LOC643031) | 1.399(0.4600) | 1.687(0.0241) | 1.206 |
| CHST15 | carbohydrate (N-acetylgalactosamine 4-sulfate 6-O) sulfotransferase 15 (CHST15) | 1.343(0.0188) | 1.523(0.0085) | 1.133 |
| APLN | apelin (APLN) | 1.854(0.1546) | 2.067(0.0458) | 1.114 |
| TOMM34 | translocase of outer mitochondrial membrane 34 (TOMM34), nuclear gene encoding mitochondrial protein | 0.686(0.0144) | 0.636(0.0014) | 0.927 |
| POP1 | processing of precursor 1, ribonuclease P/MRP subunit (S. cerevisiae) (POP1) | 0.706(0.3091) | 0.633(0.0418) | 0.896 |
| NOL6 | nucleolar protein family 6 (RNA-associated) (NOL6), transcript variant alpha | 0.745(0.0145) | 0.665(0.0488) | 0.892 |
| GPX1 | glutathione peroxidase 1 (GPX1), transcript variant 2 | 0.701(0.2286) | 0.595(0.0032) | 0.848 |
| VLDLR | very low density lipoprotein receptor (VLDLR), transcript variant 1 | 1.953(0.1747) | 1.546(0.0278) | 0.791 |
| TNFAIP8L3 | tumor necrosis factor, alpha-induced protein 8-like 3 (TNFAIP8L3) | 0.714(0.0342) | 0.559(0.0113) | 0.783 |
