# Supplementary material for: Genetic profiling of young and aged endothelial progenitor cells in hypoxia
Source: PLoS One. 2018 Apr 30;13(4):e0196572. doi: 10.1371/journal.pone.0196572 (PMC5927426; doi:10.1371/journal.pone.0196572)
Supplement: S3 Table — (DOCX) [file pone.0196572.s003.docx]

**S3 Table All genes changed by hypoxia in both young and old EPCs**

| **Gene Symbol** | **Gene Description** | **fold change (p-value), Young (hy/normal)** | **fold change (p-value), Old (hy/normal)** | **fold change (Old/Young)** |
| --- | --- | --- | --- | --- |
| ANKRD37 | ankyrin repeat domain 37 (ANKRD37) | 2.954(0.0206) | 4.870(0.0162) | 1.648 |
| VEGFC | vascular endothelial growth factor C (VEGFC) | 3.778(0.0063) | 2.515(0.0017) | 0.665 |
| HS.10862 | cDNA: FLJ23313 fis, clone HEP11919 | 3.696(0.0073) | 2.449(0.0009) | 0.662 |
| SPOCK1 | sparc/osteonectin, cwcv and kazal-like domains proteoglycan (testican) 1 (SPOCK1) | 2.917(0.0403) | 1.704(0.0226) | 0.584 |
| BHLHB2 | basic helix-loop-helix domain containing, class B, 2 (BHLHB2) | 3.927(0.0037) | 2.146(0.0007) | 0.546 |
| MGC16121 | hypothetical protein MGC16121 (MGC16121) | 1.838(0.0415) | 2.470(0.0164) | 1.343 |
| EPAS1 | endothelial PAS domain protein 1 (EPAS1) | 0.501(0.0081) | 0.664(0.0049) | 1.324 |
| ADM | adrenomedullin (ADM) | 2.925(0.0201) | 3.793(0.0029) | 1.296 |
| SEMA4B | sema domain, immunoglobulin domain (Ig), transmembrane domain (TM) and short cytoplasmic domain, (semaphorin) 4B (SEMA4B), transcript variant 1 | 1.660(0.0151) | 2.103(0.0374) | 1.266 |
| CEBPD | CCAAT/enhancer binding protein (C/EBP), delta (CEBPD) | 1.930(0.0123) | 2.192(0.0133) | 1.135 |
| FABP4 | fatty acid binding protein 4, adipocyte (FABP4) | 0.494(0.0105) | 0.560(0.0273) | 1.134 |
| NDRG1 | N-myc downstream regulated gene 1 (NDRG1) | 1.613(0.0384) | 1.748(0.0058) | 1.083 |
| HMOX1 | heme oxygenase (decycling) 1 (HMOX1) | 0.346(0.0310) | 0.369(0.0398) | 1.064 |
| LOC647886 | misc_RNA (LOC647886) | 1.613(0.0217) | 1.657(0.0131) | 1.027 |
| HTRA1 | HtrA serine peptidase 1 (HTRA1) | 1.703(0.0048) | 1.749(0.0129) | 1.026 |
| SLC2A1 | solute carrier family 2 (facilitated glucose transporter), member 1 (SLC2A1) | 4.477(0.0022) | 4.497(0.0045) | 1.004 |
| PDIA5 | protein disulfide isomerase family A, member 5 (PDIA5) | 1.521(0.0255) | 1.505(0.0167) | 0.989 |
| ENO2 | enolase 2 (gamma, neuronal) (ENO2) | 1.852(0.0375) | 1.830(0.0303) | 0.987 |
| EPB41L3 | erythrocyte membrane protein band 4.1-like 3 (EPB41L3) | 1.658(0.0406) | 1.518(0.0152) | 0.915 |
| NAV1 | neuron navigator 1 (NAV1) | 2.073(0.0009) | 1.884(0.0078) | 0.908 |
| GDF15 | growth differentiation factor 15 (GDF15) | 0.504(0.0282) | 0.439(0.0101) | 0.871 |
| SLC2A3 | solute carrier family 2 (facilitated glucose transporter), member 3 (SLC2A3) | 3.024(0.0145) | 2.491(0.0144) | 0.823 |
| GADD45B | growth arrest and DNA-damage-inducible, beta (GADD45B) | 2.025(0.0209) | 1.633(0.0131) | 0.806 |
| HSD17B2 | hydroxysteroid (17-beta) dehydrogenase 2 (HSD17B2) | 3.470(0.0273) | 2.654(0.0370) | 0.764 |
| SPAG4 | sperm associated antigen 4 (SPAG4) | 2.219(0.0012) | 1.546(0.0183) | 0.696 |
